# Evaluation of universal coverage of insecticide-treated nets in western Kenya: field surveys

**DOI:** 10.1186/1475-2875-13-351

**Published:** 2014-09-03

**Authors:** Guofa Zhou, Jessica S Li, Ednah N Ototo, Harrysone E Atieli, Andrew K Githeko, Guiyun Yan

**Affiliations:** Program in Public Health, University of California, Irvine, CA 92697 USA; Colgate University, Hamilton, New York USA; Center for Global Health Research, Kenya Medical Research Institute, Kisumu, Kenya; Kenyatta University, Thika, Kenya

## Abstract

**Background:**

Mass distribution of insecticide-treated nets (ITNs) is a cost-effective way to achieve universal coverage, but maintaining this coverage is more difficult. In addition to commonly used indicators, evaluation of universal coverage should include coverage of effective nets and changes in coverage over time.

**Methods:**

Longitudinal and cross-sectional household ITN surveys were carried out from 2010 to 2013 in six locations representing a variety of settings across western Kenya. Five indicators were used to evaluate the current status of universal coverage: 1) ITN ownership – proportion of households that own at least one ITN, 2) access index – ratio of the number of family members over the number of ITNs owned by that household, 3) operational coverage – proportion of the at-risk population potentially covered by ITNs, assuming one ITN for every two people, 4) effective coverage – population coverage of effective ITNs, and 5) usage – proportion of the population that used ITNs the previous night.

**Results:**

ITN ownership and operational coverage increased substantially from 2010 to 2013, but this increase was mostly due to the 2011 mass distribution campaign. In 2013, household ITN ownership was on average 84.4% (95% CI [78.4, 90.5]) across the six study areas, and operational coverage was 83.2% (95% CI [72.5, 93.8]). The ITN access rate was 59.1% (95% CI [56.6, 61.7]), and 40.8% (95% CI [38.3, 43.4]) of the people at risk needed more nets to achieve universal coverage. About 88.5% (95% CI [86.1, 90.9]) of the ITNs were below three years old and 16.5% (95% CI [12.1, 20.9]) of the ITNs had hole(s). The estimated effective long-lasting insecticide-treated net (LLIN) coverage was 70.5% (95 CI [58.7, 82.3]). Approximately 18.4% (95% CI [15.5, 21.4]) of the ITNs were shared by more than three persons, and the population ITN usage rate was about 75-87%. The reason for not using ITNs was almost exclusively “net not available”.

**Conclusion:**

Current methods of delivering ITNs, i.e., one mass campaign every five years and regular distribution of ITNs from health center can barely maintain the current effective coverage. Inaccessibility and loss of physical integrity of ITNs are major hindrances to achieving and maintaining universal coverage.

**Electronic supplementary material:**

The online version of this article (doi:10.1186/1475-2875-13-351) contains supplementary material, which is available to authorized users.

## Background

The World Health Organization (WHO) estimates that 3.4 billion people are at risk of malaria. Malaria caused 207 million clinical cases annually and 627,000 deaths in 2012 [1–4]. Approximately 80% of malaria cases and 90% of deaths occur in sub-Saharan Africa, and 77% of deaths in 2012 were children under five years of age [4]. To battle against malaria, WHO launched the Global Strategy for Malaria Control and the Roll Back Malaria programme in 1998. The President’s Malaria Initiative and the Global Fund to Fight AIDS, Tuberculosis and Malaria have also significantly strengthened malaria control efforts in sub-Saharan Africa [3–6].

Insecticide-treated nets (ITNs, including long-lasting insecticide-treated nets or LLINs unless otherwise specified), indoor residual spray (IRS), and artemisinin-based combination therapy (ACT) are the central components of current malaria control campaigns [2–4]. As a result of intensified malaria control campaigns during the past decade, 59 of the 103 countries that had malaria transmission in 2000 are now underway to meet the United Nations’ Millennium Development Goal target of reducing malaria incidence by 2015 [4]. The estimated malaria mortality rate has been dropped by 45% in all age groups and by 51% in children under five years of age between 2000 and 2012 [4]. Encouraged by such remarkable progress, WHO has recently set malaria elimination and eradication as the new goals in its malaria strategy [7–9]. However, the impact of current malaria control strategies could be short-lived given that insecticide resistance in the mosquito vectors and anti-malarial drug resistance in the malaria parasites are known to become more prevalent [10, 11]. The 2005 World Health Assembly (WHA) set targets of ≥80% coverage for four key interventions, including ITNs [1]. Because of the effectiveness of LLINs in malaria prevention, WHO recommended universal coverage (defined as one LLIN per two persons) of LLINs in 2007 [12–15]. More importantly, there is a need for continuous supply of new ITNs/LLINs to replace those that are torn or show waning efficacy, so as to sustain high levels of coverage to effectively reduce malaria transmission in sub-Saharan Africa [16].

Kenya is one of the malaria-endemic countries in sub-Saharan Africa with highly intense malaria transmission. In some areas of western Kenya the malaria infection rate is up to ~50% in schoolchildren [11]. ITNs have been used in Kenya since the 1980s, but coverage remained low through the early 2000s [4, 11, 17]. However, with financial assistance from the Global Fund to Fight AIDS, Tuberculosis, and Malaria, the 2006 LLIN mass distribution campaign distributed about seven million ITNs, of which 6.7 million were LLINs, targeting children under five years of age and pregnant women. ITN ownership increased from 5.9% in 2003 to 50.2% in 2006 [4, 11, 17]. In 2009, the Government of Kenya began to strive “*Towards a malaria-free Kenya*”, in response to the decreasing malaria burden and increasing malaria control efforts [17]. In 2011, a second mass ITN distribution campaign delivered 11 million LLINs free of charge as part of the efforts to achieve universal coverage of all people at risk of malaria [4]. These centralized mass distribution campaigns have served as the cornerstone of efforts to achieve universal ITN coverage. They have been shown effective at broadening household ownership of LLINs, and the proportion of households with at least one LLIN has increased substantially [17–22]. However, it is not clear how far we are from the goal of universal coverage in western Kenya. In this study, we asked what proportion of these nets is functionally effective or in good condition and what the actual rate is of ITN usage [23, 24]. We evaluated the current coverage, condition, and usage of ITNs in western Kenya with the goal to provide guidance or suggestions to public health policies in pursuit of universal access, coverage and usage.

## Methods

**Figure 1:**
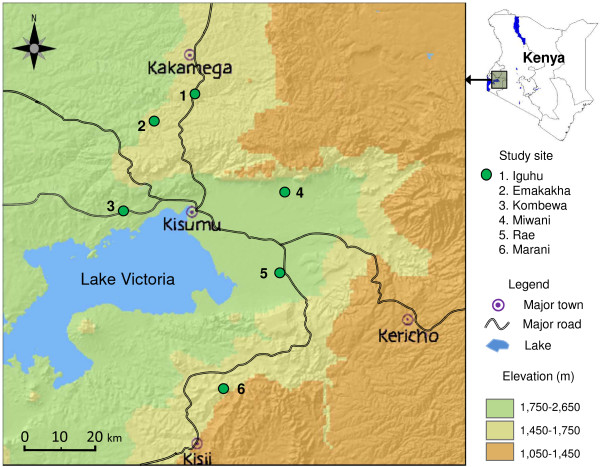
**Elevation map and study sites in western Kenya.**

This study was conducted in six study sites in western Kenya from 2010 to 2013 (Figure 1): Iguhu in Kakamega County; Emakakha in Vihiga County; Kombewa, Rae and Miwani in Kisumu County; and Marani in Kisii County. These six sites were chosen to represent different ecological, entomological and epidemiological settings across western Kenya (Additional file 1: Table S1). They include both high- and low-transmission sites, highlands and lowlands, and sites with mainly *Anopheles gambiae sensu stricto*, *Anopheles arabiensis*, *Anopheles funestus* or a mix of more than one species (Additional file 1: Table S1) [11, 25–30]. Field surveys of ITN ownership and usage were conducted along with monthly entomological surveillance. This arrangement saved time and money and avoided logistical issues such as transportation and manpower.

Houses at each site were randomly selected for participation in this study [11, 24–26]. Owners of the selected houses were requested to sign a freely administered, informed consent form covering participation in the study, questionnaire surveys and monitoring of ITN conditions. Households were interviewed about the type of nets used, the number of nets of each type and the condition of each net (e g, did the net have any holes and, if so, how many). The condition of nets was confirmed by a field technician. Households were also asked for the number, age and gender of family members and to identify which individuals slept under bed nets the night before the survey. Monthly surveys at each site were pooled as annual data for further analysis. The ITN usage surveys were completed in 2013 using cross-sectional random sampling surveys at two sites, Iguhu and Kombewa, to represent different ecological, entomological and epidemiological setting (Figure 1, Additional file 1: Table S1) [11, 24–26]. Six cross-sectional, random sampling pilot surveys were conducted in different years and at different locations to evaluate the accuracy of the monthly surveys.

Five indicators were evaluated: household ITN ownership, household access to ITN, operational coverage, effective coverage, and individual usage. Four of these indicators are recommended by WHO for the evaluation of universal coverage. Effective coverage was added as an additional indicator, which is considered to be more important than operational coverage, because if a net is not effective for prevention purposes, it is rendered useless [16]. ITN ownership is defined as the proportion of households owning at least one ITN; the rate of access to ITN is defined as the proportion of households with at least one ITN for every two people; operational coverage is defined as the proportion of the at-risk population potentially covered by ITNs, assuming one ITN for every two people by WHO standard [16]; and, usage is defined as the proportion of the population that used ITNs the previous night. Usage was further divided into age groups. Effective coverage is defined as coverage of effective ITNs, i e, ITNs in functionally good condition. A new indicator, access index (*AI*), is proposed to estimate the population in need of ITNs. *AI* is defined as the ratio of the number of family members over the number of ITNs owned by that household, assuming one ITN covers two people. A value of *AI* >2 means insufficient nets by WHO standard [16], 1 < *AI* ≤2 means sufficient, and *AI* <1 means over-covered, i e, the household has more than one net per person – they probably have more nets than they need.

Year-to-year change in average ITN ownership and average coverage across study sites was tested using t-test. Year-to-year change in ITN ownership and coverage at given study site was tested using χ^2^-test.

The number of sampled households was calculated based on an expected ITN ownership rate of 70% and precision level of 7% (10% relative error) [11]. The binomial model was used to estimate the 95% confidence interval. The total number of households in each study site was estimated to be 3,000. The sample size obtained was 165 households per site per year. Taking into consideration missing information, assuming 10% loss, the sample size used in this study was 200 households per site per year.

### Scientific and ethical clearance

Scientific and ethical clearance was given by the Kenya Medical Research Institute and University of California at Irvine institutional review boards. Volunteers were enrolled from the primary schools in the study sites through the primary school administrators with the permission of the division office of the Ministry of Health. Written assent for children (<18 years of age) were obtained by the participants and their parents or guardians. Written assent for household was obtained by the head of the household. Inclusion criteria included: provision of informed consent and no reported chronic or acute illness except malaria. Exclusion criteria include: those who were unwilling to participate in the study. According to the standard malaria treatment guidelines of the Ministry of Health of Kenya, asymptomatic infections were not treated with anti-malarials, but symptomatic volunteers were referred to the local government hospitals or clinics for diagnosis and treatment free of charge.

## Results

### ITN ownership and coverage

Over the four-year study period, 6,878 households were surveyed, of which 5,420 (78.8%) reported owning at least one ITN. With a total number of 7,888 ITNs/LLINs reported and 21,703 individuals surveyed, the overall ITN coverage was 72.7%. Operational ITN coverage increased from an average of 46.0% (95 CI [37.2, 54.9]) in 2010 to 69.7 (95 CI [68.6, 70.8]), 77.0 (95 CI [67.1, 87.0]) and 83.2% (95 CI [72.5, 93.8]) in 2011, 2012 and 2013, respectively. Year-to-year increases in average operational coverage were significant at level of 5% for 2010–2011, 2011–2012, but not for 2012–2013.

**Figure 2:**
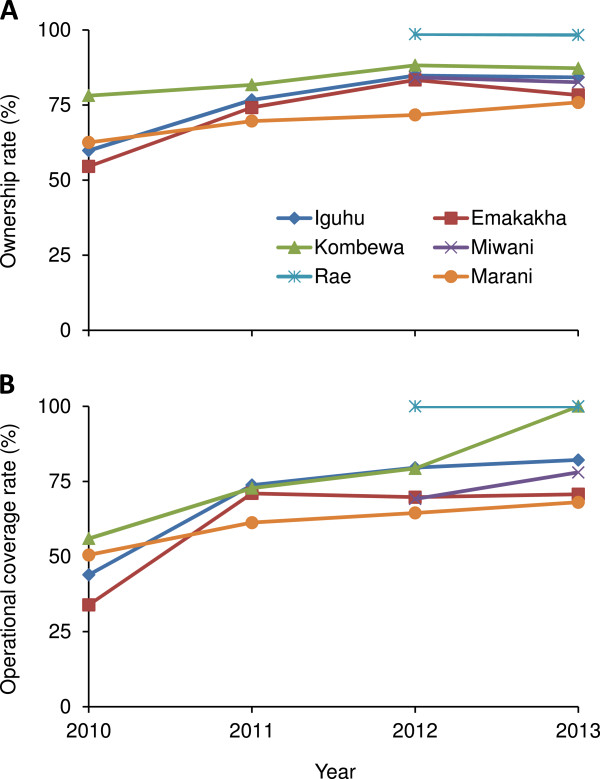
**Changes in insecticide-treated nets ownership and operational coverage in different study sites from 2010 to 2013. A**: ITN ownership, and **B**: operational ITN coverage.

ITN ownership and coverage varied among study sites but increased in all study sites since 2010 (Figure 2A). ITN ownership increased by about 20% in Iguhu (χ^2^ = 4.1, d.f. = 1, *P* = 0.042) and Emakakha (χ^2^ = 4.5, d.f. = 1, *P* = 0.034) from 2010 to 2011 while it increased by <10% in Kombewa (χ^2^ = 0.2, d.f. = 1, *P* = 0.698) and Marani (χ^2^ = 0.8, d.f. = 1, *P* = 0.372). ITN coverage increased by >30% in Iguhu (χ^2^ = 48.1, d.f. = 1, *P* < 0.0001) and Emakakha (χ^2^ = 94.6, d.f. = 1, *P* < 0.0001) from 2010 to 2011 while it increased by about 15% in Kombewa (χ^2^ = 13.9, d.f. = 1, *P* < 0.001) and Marani (χ^2^ = 7.4, d.f. = 1, *P* = 0.006) (Figure 2B). The changes in ITN ownership and coverage were insignificant from 2012 to 2013 in all study sites (χ^2^ -test, d.f. = 1, *P* > 0.05) except coverage in Kombewa (χ^2^ = 35.1, d.f. = 1, *P* < 0.0001).

**Figure 3:**
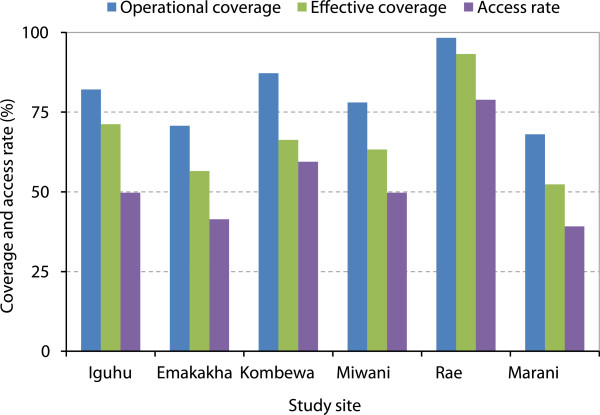
**Comparison of operational coverage, effective coverage and insecticide-treated nets access rate among different sites in 2013.**

Access to ITNs reflects the potential true coverage. In 2013, the household access rates were <50% in four out of the six sites surveyed (Figure 3). The highest household access rate was 78.9% in Rae and the lowest was 39.2% in Marani.

Pilot studies surveyed 2,897 households with a population of 10,663. ITN ownership and operational coverage were similar between monthly surveys and cross-sectional surveys (Additional file 2: Table S2). Differences in ITN ownership and operational coverage between monthly surveys and cross-sectional surveys at each survey occasion and each site ranged from 0.7 to 5.8%.

### ITN types, conditions and effective coverage

Overall, about 1.8% of ITNs surveyed in 2013 were regular ITNs and the rest were LLINs. Kombewa had the highest rate of regular ITNs at 5.9%; all other sites had rates <1.1%.

On average, 16.5% (95% CI [12.1, 20.9]) of the ITNs surveyed had at least one hole (number of holes ranging from one to 20), with the lowest rate of 5.2% in Rae and the highest rate of 22.8% in Marani. Overall, 14.8% (95% CI [13.5, 16.1]) of the LLINs and 53.8% (95% CI [49.2, 58.5]) of the regular ITNs had holes. Of the regular ITNs, 75% in Emakakha and 40.7% in Kombewa had holes, and 100% of the regular ITNs in the other sites had holes.

Taking into consideration ITN ownership, operational coverage, types and conditions, the estimated effective LLIN coverage was on average 70.5% (95% CI [58.7, 82.3]). The highest effective coverage was 95.1% in Rae and the lowest was 57.3% in Marani (Figure 3).

### ITN usage

In 2013, the ITN usage rate was 87.5% in Kombewa and 75.2% in Iguhu. Usage rates were about 10% lower than ownership and operational coverage rates (Table 1). Young children (zero to four years) had the highest usage rate and older children (five to 14 years) had the lowest usage rate (Table 1).

**Table 1:** **Insecticide-treated net ownership and usage in Kombewa and Iguhu in 2013**

Parameter	Age group	Kombewa	Iguhu
ITN ownership (%)		97.5 (195/200)	86.6 (174/201)
Operational coverage (%)		100 (354x2/701)	87.9 (351x2/799)
ITN usage (%) by age group			
	0-4	98.8 (88/89)	81.1 (99/122)
	5-14	80.7 (184/228)	70.0 (170/243)
	≥15	88.8 (341/384)	76.5 (332/434)
	Total	87.5 (613/701)	75.2 (601/799)

**Figure 4:**
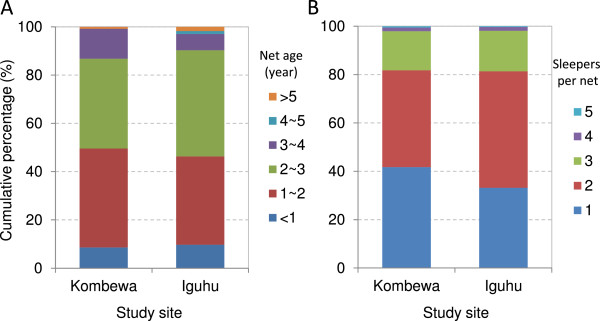
**Distribution of net age and number of sleepers per net in Iguhu and Kombewa in 2013. A**: distribution of net age, and **B**: distribution of number of sleepers per.

Less than 10% of the nets were obtained during the last 12 months and about 10% of the nets were older than three years (Figure 4A), while about 1% of the nets were older than five years. While over 80% of nets had one or two sleepers, which is considered appropriate coverage, the rest had more than two sleepers and about 2% had four to five sleepers (Figure 4B). Overall, 40.8% (580/1,419) of the individuals surveyed had insufficient ITN coverage (Table 2). Interestingly, 17 households had reserved 26 nets for future use. The reason given for not using ITNs was almost exclusively “net not available” (>99%); less than 1% of households responded with “not enough funds”.

**Table 2:** **ccessibility of insecticide-treated nets by household and proportion of people needing nets in 2013**

Parameter		Access index	Number of households	Number of ITNs	N individuals	
					Total	Need net (%)
Household without ITN		n/c†	28	0	92	92 (100)
With ITN but not all individuals covered		3.5	76	104	348	146 (42)
With ITN and all individuals covered						
	Over-covered	<1	10	22	19	0
	Sufficient coverage	1–2	197	424	618	0
	Insufficient coverage	> 2	71	127	342	342 (100)
Total		2.1	382	677	1,419	580 (41)

†Not calculated.

Pooled surveys of Kombewa and Iguhu.

## Discussion

ITNs have proven to be one of the most effective tools for malaria control [31–34]. The scaling-up of ITNs, together with IRS and ACT, has tremendously reduced malaria infections all over the world during the last decade [3]. WHO has set universal coverage, defined as full coverage with effective vector control, as the goal for all people at risk of malaria [4]. Mass distribution campaigns are a cost-effective way to rapidly achieve high and equitable ITN coverage in at-risk populations. WHO recommends four indicators for the evaluation of universal coverage: a) percentage of households with at least one ITN/LLIN; b) percentage of population with access to an ITN/LLIN within the household; c) percentage of population reporting having slept last night under an ITN/LLIN; and, d) percentage of children under five reporting having slept last night under an ITN/LLIN [16]. In addition to these cross-sectional outcome indicators, WHO also recommends that other indicators, such as changes in coverage over time, are likely to be necessary in order to manage operations. But effective coverage, i e, coverage of effective ITNs, is not among the recommended indicators. In order to maintain universal coverage, WHO recommends that countries should apply a combination of mass free distributions and continuous distributions through multiple channels, in particular antenatal and immunization services. In Kenya, free ITNs are distributed through antenatal services in government-run public health centres.

Compared to studies carried out in western Kenya in 2009, ITN usage, especially among children under five years old, has increased tremendously [24]. This is partly due to the increased availability of ITNs, especially after the 2011 mass campaign; it is also due to increased awareness of the benefits of using ITNs, as demonstrated by the shrinking gap between ITN ownership and usage rates. Compared to studies conducted in other malaria-endemic countries [18–22, 35], ITN usage in the general population was higher in this study, and the gap between ITN ownership and usage rates was smaller. The higher proportion of people using nets may imply that western Kenyans have higher ITN access rates than populations in other countries.

Two crucial points need to be highlighted from this study: the importance of effective coverage and of the maintenance of universal coverage. Effective coverage is the key to preventing mosquito bites and malaria infections, and is defined as the coverage of effective ITNs. Although non-insecticide-treated nets and ITNs that have lost efficacy may prevent mosquito bites, effective ITNs can kill mosquitoes, adding to their impact in reducing malaria transmission [36]. Mass distribution campaigns can quickly increase ITN coverage, but coverage gaps start to appear almost immediately post-campaign through net deterioration, loss of nets and population growth; therefore, continuous distribution channels are required to complement the mass campaigns because rates of declined physical integrity in LLINs can be high, and preventive efficacy can be greatly compromised [23, 36]. In this study, about one out of six ITNs had hole(s) even though most ITNs were < three years old. Although nets with small holes may still be protective, but their effectiveness may be reduced [37]. Therefore, effectiveness/effective coverage of ITNs should be considered as an important indicator when evaluating universal coverage after recent mass distribution campaigns [18–22, 35–38].

Maintaining coverage can be achieved through continuous distribution of ITNs, but it is difficult to fully close the gap in universal access to ITNs. Although ITN ownership and coverage rates are high in western Kenya, only half of the households have enough nets to cover all family members, and over 40% of the population needs more ITNs in order to achieve universal coverage. Because of the unavailability of ITNs, more than two people may be forced to share one net, which may reduce the protectiveness. Although net users in Africa may have a different view on personal space, in addition, small infants or young children sharing a net with parents is understandable, practically more than two adults sharing one net signifies a shortage of nets. Indeed, WHO guidelines used “two persons one net” as baseline assumption when evaluate the effectiveness of ITN campaigns. Furthermore, it is difficult to monitor the effectiveness of ITNs. In this study, one out of six ITNs had at least one hole, meaning that presumably the nets are not fully effective and need to be replaced. Considering 98% of the ITNs are < four years old, the average annual loss of ITNs due to being torn is approximately 5%. The lifespan of ITNs varies widely between individual nets in a cohort and between settings. In several settings in Africa, the median lifespan (the interval until 50% of the nets are worn out or lost) of a cohort of LLINs has been observed to be approximately three years [16]. Using 50% and a three-year lifespan as the cut-off, the percentage of ITNs needing to be replaced in western Kenya would be 11% annually.

In Kenya, ITNs are freely distributed by government-run health centres. There is a limited number of health centres, and additional free nets are usually distributed only to pregnant women who present at the health centres. Therefore, households without pregnant women but needing additional nets are missed. In the study areas, the increase in ITN coverage on average was 24, 7, and 6% in 2011, 2012, and 2013, respectively. This means that the current practice – one mass campaign every three years plus regular health centre distribution of ITNs – can barely maintain the current effective coverage.

## Conclusion

Current methods of delivering ITNs – one mass campaign every five years plus regular health centre distribution of ITNs – can only maintain the current effective coverage. Inaccessibility and loss of physical integrity of ITNs are the major barriers to achieving and maintaining universal coverage. Therefore, to attain and sustain universal access and coverage, additional distribution channels need to be researched and new distribution methods need to be implemented.

## Electronic supplementary material

Additional file 1: Table S1: Study site background information. Description: Information about the elevation and ecological, entomological and epidemiological characteristics of the study site. (DOCX 19 KB)

Additional file 2: Table S2: Comparison in ownership rate and operational coverage rate between monthly surveys and cross-sectional surveys (pilot in the table) in different sites at different survey occasion. Description: Comparison in ownership rate and operational coverage rate between monthly surveys and cross-sectional survey (pilot in the table) in different sites at different survey occasion. Cross-sectional survey was conducted once a year in July. Number of households surveyed is the total number over the 12 months. (DOCX 17 KB)
