# Supplementary material for: Evaluation of universal coverage of insecticide-treated nets in western Kenya: field surveys
Source: Malar J. 2014 Sep 3;13:351. doi: 10.1186/1475-2875-13-351 (PMC4162923; doi:10.1186/1475-2875-13-351)
Supplement: Supplementary file 1 — Additional file 1: Table S1: Study site background information. Description: Information about the elevation and ecological, entomological and epidemiological characteristics of the study site. (DOCX 19 KB) [file 12936_2014_3384_MOESM1_ESM.docx]

Additional file 1

Table S1. Study site background information.

| Site | Altitude (m) | 2001 - 2013 annual average ^†^ | | | |  | 2013 monthly average | |  | Anopheles mosquito in 2013 (%) ^‡^ | | | Major crop (>80) |
| --- | --- | --- | --- | --- | --- | --- | --- | --- | --- | --- | --- | --- | --- |
|  |  | Temperature (°C) | | | Rainfall (mm) |  | Mosquito density ^#^ | Parasite prevalence (%) ^§^ |  | *An. gambiae* s.s. | *An. arabiensis* | *An. funestus* |  |
|  |  | Max | Mean | Min |  |  |  |  |  |  |  |  |  |
| Rae | 1160-1180 | 29.8 | 23.3 | 17.5 | 1471 |  | 1.44 | 25.9 |  | 0 | 81.2 | 18.8 | Rice |
| Miwani | 1180-1220 |  |  |  |  |  | 1.17 | 28.0 |  | 0 | 41.8 | 58.2 | Rice |
| Kombewa | 1210-1320 |  |  |  |  |  | 1.49 | 57.7 |  | 28.5 | 3.7 | 67.8 | Maize |
| Iguhu | 1430-1580 | 26.9 | 20.5 | 14.9 | 2013 |  | 0.76 | 9.3 |  | 52.8 | 5.3 | 41.9 | Maize |
| Emakakha | 1450-1530 |  |  |  |  |  | 0.73 | 27.0 |  | 66.4 | 0 | 33.6 | Maize |
| Marani | 1540-1760 | 25.4 | 20.1 | 15.8 | 2025 |  | 0.45 | 0.5 |  | 16.9 | 0 | 83.1 | Maize |

^†^ Meteorological station data, Kisumu station for Rae, Miwani and Kombewa; Kakamega station for Iguhu and Emakaha; and Kisii station for Marani.

^#^ Pyrethrum spray catch method, density unit is female/house/night [11].

^§^ Parasite prevalence was estimated from school children 6-13 years old [11].

^‡^ Species composition in 2013. Proportion of *An. funestus* was calculated using 2013 data, species composition of *An. gambiae* s.l. complex was estimated using 2013 data following 2010 PCR results [11,28].
