# Supplementary material for: Evaluation of universal coverage of insecticide-treated nets in western Kenya: field surveys
Source: Malar J. 2014 Sep 3;13:351. doi: 10.1186/1475-2875-13-351 (PMC4162923; doi:10.1186/1475-2875-13-351)
Supplement: Supplementary file 2 — Additional file 2: Table S2: Comparison in ownership rate and operational coverage rate between monthly surveys and cross-sectional surveys (pilot in the table) in different sites at different survey occasion. Description: Comparison in ownership rate and operational coverage rate between monthly surveys and cross-sectional survey (pilot in the table) in different sites at different survey occasion. Cross-sectional survey was conducted once a year in July. Number of households surveyed is the total number over the 12 months. (DOCX 17 KB) [file 12936_2014_3384_MOESM2_ESM.docx]

Additional Table S2. Comparison of ownership rate and operational coverage rate between monthly surveys and cross-sectional survey (pilot in the table) in different sites at different survey occasion. Cross-sectional survey was conducted once a year in July. Number of households surveyed during monthly survey is total number of households surveyed over the 12 months.

| Study site | Year and Survey type | Number of households | | Total number of nets | Population surveyed | | Ownership rate | Operational coverage |
| --- | --- | --- | --- | --- | --- | --- | --- | --- |
|  |  | Surveyed | Own nets |  | Adults | Children |  |  |
| Emakakha | 2010 monthly | 354 | 193 | 248 | 630 | 835 | 54.5 | 33.9 |
|  | 2010 pilot | 425 | 237 | 283 | 729 | 974 | 55.8 | 33.2 |
|  | 2011 monthly | 178 | 132 | 268 | 331 | 424 | 74.2 | 71.0 |
|  | 2011 pilot | 849 | 675 | 1230 | 1623 | 1849 | 79.5 | 70.9 |
| Iguhu | 2010 monthly | 336 | 201 | 225 | 485 | 540 | 59.8 | 43.9 |
|  | 2010 pilot | 739 | 401 | 488 | 1095 | 1148 | 54.3 | 43.5 |
|  | 2011 monthly | 330 | 253 | 356 | 521 | 444 | 76.7 | 73.8 |
|  | 2011 pilot | 483 | 378 | 612 | 810 | 935 | 78.3 | 70.1 |
|  | 2013 monthly | 361 | 304 | 434 | 593 | 464 | 84.2 | 82.1 |
|  | 2013 pilot | 201 | 174 | 351 | 434 | 365 | 86.6 | 87.9 |
| Kombewa | 2013 monthly | 360 | 334 | 548 | 483 | 457 | 92.8 | 100.0 |
|  | 2013 pilot | 200 | 193 | 354 | 384 | 317 | 96.5 | 100.0 |
